# Resilience in the Face of Disruption: Viewpoint on the CrowdStrike Incident in July 2024

**DOI:** 10.2196/69958

**Published:** 2025-09-02

**Authors:** Christopher R Dennis, Christopher S Evans, Kathleen Duckworth, Misty McLawhorn Skinner, John Hanna, Tanya Thompson, Donette Herring, Richard J Medford

**Affiliations:** 1 Department of Information Services, ECU Health, 2190 Beasley Drive, Greenville, North Carolina, 27834, United States, 1 252-847-4133, 1 252-847-5561

**Keywords:** computer security, clinical informatics, health information systems, incident response, incident command system, crowdstrike health IT disruption, IT service management, system crash recovery

## Abstract

In an era where health care is increasingly dependent on digital infrastructure, the resilience of health IT systems has become a cornerstone of patient safety and operational continuity. As cyber threats grow in frequency and sophistication, health care organizations have turned to advanced cybersecurity tools to safeguard their systems. Yet even the most robust defenses can falter. On July 19, 2024, a routine update from a widely used cybersecurity platform triggered a widespread IT disruption. A flawed sensor configuration led to 8647 “blue screen of death” (BSOD) events, with 729 devices requiring manual remediation. What unfolded was not just a technical crisis but a test of organizational agility, collaboration, and resilience. This viewpoint traces the response to that disruption, highlighting the pivotal role of clinical informaticists and the coordinated efforts that enabled a rapid recovery. From the formation of an incident response team to the triage and mitigation of impacted systems, the response was swift and strategic. Clinical informaticists emerged as key players, bridging the gap between technical teams and frontline care providers. They identified workflow disruptions, facilitated communication, and ensured that patient care remained as uninterrupted as possible. Despite the scale of the outage, operations continued with minimal disruption—thanks to early recognition, decisive action, and cross-disciplinary collaboration. This incident underscored the importance of a well-practiced response plan, clear communication channels, and the integration of clinical expertise in technical recovery efforts. As we reflect on this event, several lessons emerge: the need for continuous refinement of incident response strategies, the value of regular training exercises, and the critical role of clinical informatics in navigating digital crises. This paper calls for a renewed commitment to building resilient health IT ecosystems—ones that can withstand disruption and continue to support the delivery of safe, effective care.

## Introduction

Cybersecurity and the related disruptions to critical IT systems are a major concern for many organizations due to risks associated with data breaches. This is particularly true in health care organizations due to their unique risk profiles. Health care facilities are high-value targets due to their size, technological dependence, sensitive data, and vulnerability to disruptions [1]. Furthermore, there has been an increase in cyberattacks in recent years, as evidenced by a 93% increase in large breaches reported by the Office for Civil Rights under the Department of Health and Human Services between 2018 and 2022, many involving ransomware attacks [1].

The health care industry continues to deal with a barrage of attacks, with over 1400 cyberattacks globally per week [2]. The range of attack strategies used by threat actors includes social engineering, phishing, distributed denial of service, botnets, zero-day exploits, person-in-the-middle, malware, adware, and ransomware [3]. When hospitals are forced to implement downtime procedures as a result of a successful attack, the average duration is upwards of 24 days, at an average cost of US $10 million [2]. There are additional risks beyond the direct impacts to systems and the operations of the affected organizations. Cyberattacks often result in data breaches that can impact patients receiving care at the affected institutions. In 2024, over 250 million individuals were affected by health care security breaches, with 190 million of those involving a ransomware attack on the Change Healthcare network [4]. Health care entities that endure these kinds of attacks may also incur legal ramifications and reputational damage in addition to the financial impacts [3]. The legal ramifications include violations related to the Health Insurance Portability and Accountability Act (HIPAA), since the presence of any type of malware on a covered entity’s or business associate’s computer is considered a security incident under the HIPAA security rule [5]. Compromised patient data can be misused for identity theft, blackmail, and even insurance fraud. Even if the threat actor does not use the information directly, it can still be disseminated on illegal forums, further increasing the risk of misuse [3].

Defense against malicious actors requires continuous vigilance and system hardening against potential attack vectors. The Center for Internet Security publishes best practices called Critical Security Controls (current version, as of this writing, is version 8.1), which detail the steps organizations can take to guard against attacks [6]. CrowdStrike is a leading cybersecurity firm that provides services to many organizations, both inside and outside of the health care sector, to achieve these goals. Among these services is the Falcon platform, which is designed to stop breaches and protect organizations from a variety of cybersecurity threats.

On July 19, 2024, at 04:09 UTC (12:09 AM local time), CrowdStrike released a sensor configuration update to Microsoft Windows systems to maintain the protection mechanisms of the Falcon platform. This update triggered a logic error, which resulted in a system crash and “blue screen of death” (BSOD) on impacted systems. This error was quickly identified by the team at CrowdStrike, and the sensor configuration update was swiftly remediated on July 19, 2024, at 05:27 UTC (1:27 AM local time). The specific machines that were impacted needed to be running the Falcon sensor for Microsoft Windows versions 7.11 and above, be online between July 19, 2024, 04:09 UTC and July 19, 2024, 05:27 UTC, and have downloaded the flawed configuration update during that interval [7].

Importantly, the system failure that occurred in this case was not the result of a cyberattack, but the impacts to critical systems and the recovery strategies used shared similarities with those that would be enacted if systems had been compromised due to a breach. While the underlying cause of the logic error was quickly identified and remediated by CrowdStrike, the downstream impacts to customers were substantial and required a coordinated strategy to mitigate.

Many health care workflows are dependent upon seamless integration of otherwise disparate systems. For example, radiology practices require coordination between imaging devices, a Picture Archiving and Communication System, and the electronic health record (EHR). Each of these components represents an independent point of failure in the event of a major health IT outage, such as the one encountered with the CrowdStrike incident [8]. Considering the time-sensitive nature of significant disruptions in health IT, it is crucial to have clear and effective communication processes, especially in large, geographically dispersed health care systems that rely on health IT infrastructure for patient care and operations.

The CrowdStrike incident highlighted the significant risks associated with workstation downtime, including medication errors, inaccessibility of images and test results, and the need to delay procedures. During the incident, many health care workflows were disrupted due to the inability to access critical patient information on affected workstations (eg, radiology images). In addition, the inability to access lab results on time further compounded the delays in diagnosing and treating patients. Although the EHR itself remained operational, the downtime of numerous workstations could have led to delays in reviewing and acting upon patient information, ultimately impacting the quality of care provided. These risks are well-documented in literature, emphasizing the importance of proactive planning for EHR downtime [9-12].

The primary objective of this viewpoint is to offer real-world insights following a major IT disruption by exploring ECU Health’s response to the CrowdStrike incident. In addition, the authors share lessons learned from leveraging a multidisciplinary team across Information Services (IS), including a well-established network of embedded clinical informaticists, to navigate the response efforts. Finally, we describe opportunities for improvement that should help similar organizations address similar challenges in the future.

## Background

ECU Health is a rural academic health care system that serves 29 counties in eastern North Carolina. The health care system comprises 9 hospitals spanning in size and scope from a large academic level 1 trauma center to critical access hospitals. Within this network are 185 primary care and specialty clinics in 110 locations, plus other outpatient facilities, home health, hospice, and wellness centers [13]. Together, these ambulatory practices complete over 800,000 visits annually. All ECU Health facilities are served by an enterprise-wide IS division, which oversees all aspects of health IT, including hardware, software (eg, EHR and clinical applications), networking, end-user support (EUS), and cybersecurity.

The clinical informatics team within the IS division, consisting of 19 full-time informaticists and 7 full-time informatics education specialists with diverse clinical and nonclinical expertise, serves as a critical interface between clinical teams, operational leaders, and health IT professionals. These informaticists integrate health care sciences, computer science, and information science to manage and communicate data, information, and knowledge, thereby enhancing patient care, improving health outcomes, and supporting decision-making processes [14,15].

## Existing Incident Response Strategy

ECU Health’s incident response strategy includes the formation of the incident response team, initial assessment and triage of the situation, and identifying key actions to mitigate impact. When an IS team member is notified of an incident affecting multiple users, the first escalation is to the IT service management team for vetting and validation. The incident is next escalated to the IS executive on call, who determines whether it should be categorized as a Priority 1 (P1)–critical outage. Incidents are categorized according to their urgency and impact, as shown in Table 1. Patient care incidents are prioritized higher than nonpatient care issues. If categorized as a P1, a major incident is created within the enterprise ticket management solution, and the operational executives on call for the impacted facilities are notified. A bridge line (ie, a dedicated conference call line for coordinating team members) is opened, and command centers are mobilized at the impacted sites. The command center at the medical center serves as the central hub for local hospital operations and as a physical location for the systemwide command center. The systemwide command center is staffed with IS and operational leaders. Regional command centers are staffed with local operational leaders. Each command center’s primary roles are to centralize communications, facilitate issue escalation, and disseminate updates as issues are corrected and functionality is restored. Additional bridge lines are opened within IS, including a technical one to problem-solve issues and determine impacted applications.

**Table 1.** Impact and urgency matrix used to categorize incidents.

Urgency	Hospital or organization-wide	Multiple users	Single user: patient care	Single user: nonpatient care
Critical	Critical (P1)^a^	Critical (P1)	High (P2)^b^	Medium (P3)^c^
High	Critical (P1)	High (P2)	Medium (P3)	Medium (P3)
Medium	High (P2)	Medium (P3)	Medium (P3)	Low (P4)^d^
Low	Medium (P3)	Low (P4)	Low (P4)	Low (P4)

a P1:priority 1.

b P2:priority 2.

c P3:priority 3.

d P4:priority 4.

During the initial assessment and triage phase, the IS command center relies on bridge line communication to determine the scope of impacted applications and incident tracking. All incidents are tracked through the ticket management system. As new incidents are reported, they are tracked as “children” of the “parent” incident if found to be related.

Finally, actions are taken to address the situation, including isolating the impacted systems, communicating with staff and patients, and initiating interventions to correct the underlying problems or implementing short-term alternatives. These measures ensure that the immediate risks are mitigated and that all stakeholders are informed about the ongoing efforts. By isolating the affected systems, further spread of the issue is prevented, while clear communication helps maintain trust and transparency. Timely initial interventions are crucial for stabilizing the situation and providing temporary solutions until a permanent fix can be implemented. This comprehensive approach helps manage the incident effectively and minimizes disruptions to operations.

## Timeline of Events, Organizational Impacts, and Remediation Efforts

Following the CrowdStrike update, initial reports of devices receiving the BSOD began on July 19 at 1:30 AM. The sequence of events from the initial BSOD reports to the closure of the systemwide command center approximately 30 hours later is shown in Table 2. 

**Table 2.** ECU Health event response timeline for the CrowdStrike incident.

Time	Event
Friday, July 19	
12:09 AM	CrowdStrike releases flawed sensor configuration update
1:27 AM	CrowdStrike remediates the flawed sensor configuration update
1:30 AM	Initial reports of workstations and servers receiving the BSOD^a^
1:38 AM	Initial escalation to Information Services (IS) team members
3:40 AM	Escalation to the IS executive on call and then to operational leadership
4:45 AM	Email notification sent to IS staff indicating the BSOD issue had been traced back to the CrowdStrike update
5:30 AM	The systemwide Incident Command Center was opened
6:00 AM-7:00 AM	Clinical informaticists arrive on site at hospital locations prepared for deployment
6:58 AM	Initial notification for IS personnel to report to their closest system hospital
8:20 AM	Systemwide email indicating that command centers had been activated at all system hospitals; users experiencing BSOD are encouraged to reboot their workstations twice; and for issues with ECU Health mobile devices, docking stations were to be unplugged.
10:49 AM	Staff are encouraged to submit incidents into the ticketing system for individual workstations experiencing BSOD unable to be remediated by staff directly
12:29 PM	Health care system staff informed of potential scammers posing as IT professionals attempting to gain access to computers and files
4:32 PM	Command center at the medical center converted from in-person to remote format; all community hospital command centers closed
5:00 PM	The IS meets to recap the day’s events and discuss plans for the weekend
Saturday, July 20	
8:18 AM	The medical center closes its command center

a BSOD: blue screen of death.

Across the ECU Health enterprise, there are approximately 14,500 end point devices. There were 8647 BSOD events (approximately 60% of devices) on July 19. Most of these workstations were corrected with a reboot once the remediated sensor configuration update was available. Of those that were not corrected with a reboot, 729 devices required hands-on remediation. Approximately 150 workstation thin clients (ie, machines that rely on a central server for processing power and storage) and 100 ground control workstations (ie, centralized systems used to manage, monitor, and control devices for mobile device management) were reimaged to restore them to a functional state.

There are 3000 Windows servers used across the enterprise. Of these, 700 (23%) deliver Citrix applications, including the EHR. Approximately one-third of these devices were impacted by the flawed sensor configuration update. These servers are nonpersistent, meaning they load a golden image (ie, a preconfigured, fully functional template of a computer system and software configuration) each time they are restarted, and temporary data or changes made during the previous session are discarded. There are approximately 100 other persistent servers that provide other services related to the EHR (eg, print servers).

Users working with impacted devices could not access core applications and systems, including the EHR. However, the EHR itself remained operational throughout this incident, and not all workstations were impacted. IS leadership assessed the availability of the EHR throughout the incident to guide recovery efforts. Timing was a significant factor in keeping the EHR operational, as the incident occurred during a low-user volume period, which prevented the whole fleet of servers that deliver the EHR from being engaged and encountering the BSOD conditions.

In the months preceding this incident, the health care system deployed cell phones to clinical staff to support patient care and internal communication. Many of these were not impacted and enabled workflows such as medication administration to proceed even when nearby workstations were unavailable.

Impacted devices were first rebooted. If the issue persisted, an incident was logged to identify it for hands-on support. These devices were then physically accessed by IS personnel. The devices were booted in “Safe Mode” using a BitLocker recovery key, if necessary, and the flawed channel file (C-00000291*) was manually deleted. The device was then rebooted, which enabled the system to start without the BSOD and allowed an update to the channel file to flow across to the machine. These steps are described in more detail on CrowdStrike’s website [16]. Once CrowdStrike released the remediated sensor configuration, nonpersistent servers were corrected with a reboot. For persistent servers, these were recovered using the method described above in this paragraph, which involves booting them into “Safe Mode” and removing the flawed channel file.

Some workstations required remediation using a USB drive to boot the system with Kali Linux (Offensive Security). The IS team members ensured they had adequate privileges to perform actions on the directory that contains the CrowdStrike drivers. The files within were removed, the device was unmounted, and the machine was restarted without the USB drive so that it boots normally. If these options were unsuccessful, the devices were reimaged to restore them to a functional state.

## Role of Clinical Informatics in the Recovery Efforts

As soon as clinical informatics became aware of the issue, their first course of action was to call every unit manager (or charge nurse if the unit manager was unavailable) throughout the health care system to make sure they were informed of the situation and that CIs and other IS team members would be on the units helping to troubleshoot and restore the affected devices. During those phone calls, floor staff were encouraged to self-report any machines that were known to be impacted. This was intended to expedite the discovery process, leading to a more rapid resolution. The manager of the clinical informatics team was a key contributor to the ongoing bridge line calls and kept the systemwide incident command center apprised of progress toward remediation of all devices throughout the enterprise. This enabled resources to be focused on priority areas based on the volume of workstations impacted and the propensity for delays in care if not resolved urgently.

The network of CIs was dispatched across the enterprise to their respective sites to augment remediation efforts in coordination with EUS, service desk, and other IS personnel. As a result of the collaborative relationships clinical informaticists have established with clinical operations, CIs became another contact for reporting impacted devices, particularly if there was difficulty accessing or unfamiliarity with reporting incidents. The presence of CIs also instilled a sense of calm in clinical staff, especially during the early phase of the incident. The scale of the outage, both within and outside of the organization, had the potential to create panic due to concerns that this issue was caused by a cyberattack, as expressed by some end users after perusing social media. The CIs were well-positioned to provide accurate information to staff about the nature of the incident as a faulty system update and not a cyberattack. This assuaged their fears and helped ensure staff were able to focus on completing the necessary steps to rectify the situation and bring all systems back to a functional state.

In an effort to avoid duplicate reporting, CIs used sticky notes to label impacted devices as “reported,” which helped to limit confusion and focus recovery efforts. The role of CIs in the incident response varied based on diverse needs across sites and included consistent communication, incident reporting, and at-the-elbow support. At-the-elbow support was particularly beneficial because it helped clinicians identify and execute the remediation steps when necessary while ensuring patient care remained uninterrupted.

## Key Lessons Learned

Health care organizations are dependent upon technologies to deliver patient care. Considering the scope of this outage, the risk of interruptions that could adversely impact patient care was high. Due to rapid problem recognition and mobilization of IS staff and resources, ECU Health continued operations as usual despite device outages. This speaks to the level of teamwork exhibited throughout the health care system. Even though ECU Health recovered most devices within 24 hours, there are still opportunities to refine its incident response.

Major incidents such as the CrowdStrike outage highlight the importance of having a well-prepared and accessible incident response plan using a framework such as the Hospital Incident Command System [17]. Incident response plans should include clear roles and responsibilities with clarity around decision-making. These plans should also include regular system audits, updates, and reviews to ensure applicability as health care systems continuously change.

Effective and consistent communication during disruptions is paramount. Device outages were first identified around 1:30 AM on July 19th. However, IS leaders were not notified until hours later, representing a missed opportunity for faster mobilization. ECU Health will refine its process for issuing broader alerts based on the event’s criticality. In addition, communication challenges were observed related to reliance upon different devices and modalities for call tree escalation. Risks included issues such as battery performance, devices being out of reach, or susceptibility to outage conditions. ECU Health is exploring synchronization of contact numbers with backup redundancy, such as personal devices, to ensure communication capabilities during after-hours incidents.

Dispatching CIs who have established relationships with front-line clinicians allowed for responsive and multimodal support, including at-the-elbow support and virtual troubleshooting. This was invaluable, as it would have been impossible to manage the scale of this outage with the service desk, EUS, and IS teams alone. The effectiveness of this strategy relies on the informal cross-training of clinical informaticists across IS roles during routine operations. Our experience during this major disruption demonstrated that CIs leveraged their skill sets effectively during this crisis and showed the value of cross-training across roles.

In the months leading up to this outage, ECU Health had been deploying Microsoft Teams as the enterprise collaboration and communication platform. This tool was an effective communication method during this event when other tools were unavailable. Video meeting recordings and artificial intelligence–augmented transcription allowed for rapid summaries for team members joining multiple calls simultaneously. Further investigation will be conducted to determine whether its availability was due to inherent fault tolerance or whether scenarios exist under which it may not be as reliable as it was during this event. More broadly, ECU Health has identified a need for an enterprise-wide alerting system to enable timely communication during emergencies. Minor challenges were encountered related to authentication processes that were required during the outage, indicating a need for better preparedness in handling such challenges in the future.

## Changes Going Forward

### Overview

**Figure 1.**
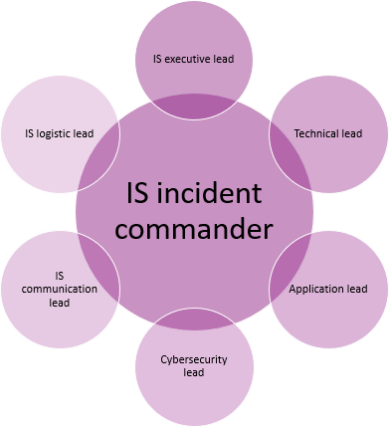
Key roles in the Information Services (IS) incident command system, showcasing the central role of the IS incident commander.

Based on the lessons learned from this event, ECU Health is undertaking several initiatives as part of its commitment to a culture of continuous improvement. One of these initiatives involves enhancements to the IS Incident Command Structure. The key roles of the enhanced IS Incident Command Structure are shown in Figure 1, and their core responsibilities are provided in the following subheadings.

### IS Incident Commander (Member of the Information Technology Service Management Team)

The IS incident commander will lead the incident response. This individual will coordinate all response workstreams to understand and communicate impacts to the IS executive lead. Other responsibilities include managing tasking for all IS workstreams to ensure proper prioritization of actions, managing shift planning to ensure adequate staffing throughout the incident, and developing situation reports (SITREPs) for proactive communication, which will provide a single source of truth on the impacts of actions taken. This individual will also review the outputs of workstreams and ensure objectives are met in a timely fashion.

### IS Executive Lead (Member of the IS Senior Leadership Team)

The IS executive lead will work closely with the IS incident commander and serve as the primary liaison between IS and senior operational leadership. This leader will be an escalation point for any major blockers that cannot be resolved by the response team leaders. Through their interactions with IS personnel and senior operational leadership, they will maintain visibility into the recovery efforts and understand the impacts of these efforts on the wider organization.

### Application Lead (IS Manager or Above)

Application leads will be assigned based on the nature of the outage and the impacted applications. Those assigned to these roles will perform an initial triage to understand the impact of the issue, ensure that people with the correct application knowledge and skill sets are engaged, and identify any relevant vendor contracts for escalation and support. As the situation evolves, they will provide updates to the IS incident commander and assist with the production of the SITREPs. They will support and guide the troubleshooting efforts, assist with tasks as necessary, and request additional resources as needs are identified. As workarounds or solutions to the problem are identified, the application lead will be responsible for specifying the testing requirements for the application to ensure successful resolution and to help anticipate potential unintended consequences.

### Technical Lead (IS Manager or Above)

Technical leads will be assigned based on the nature of the outage and the impacted systems. Those assigned to these roles will perform an initial triage to understand the impact of the issue, ensure that people with the correct technical skill sets are engaged, and identify any relevant vendor contracts for escalation and support. As the situation evolves, they will provide updates to the IS incident commander and assist with the production of the SITREPs. They will support and guide the troubleshooting efforts, assist with tasks as necessary, and request additional resources as needs are identified. As workarounds or solutions to the problem are identified, the application lead will be responsible for specifying the testing requirements to ensure successful resolution and to help anticipate potential unintended consequences.

### Communication Lead (Member of the IT Service Management Team)

The communication lead is responsible for controlling the messaging to staff throughout the enterprise. Examples of this messaging may include unscheduled downtime notices or IS outage communications. They will work with the operations communication team if responsibility for external communication needs to be delegated. Any requests for information will be triaged by this lead, and their response should be consistent with other messaging that is disseminated more broadly within the organization.

### Logistics Lead (Supervisor, Manager, or IS Team Member)

The logistics lead is responsible for coordinating and instructing on-site resources and works closely with the application and technical leads to ensure these resources are appropriately prioritizing the work to be done. This lead is responsible for both the human resources and any equipment that is necessary to resolve the incident. This lead receives updates from on-site resources and shares this information with the application and technical leads as appropriate.

### Cybersecurity Lead (Manager or Above)

The cybersecurity lead directs activities that are related to incidents stemming from cybersecurity events. This lead participates in the incident response, engages risk management and the legal team as appropriate, and ensures appropriate communication and information collection relevant to the current cybersecurity risk state. In addition to these updates to the IS incident command structure, ECU Health will conduct tabletop exercises and major incident refresher training. Tabletop exercises will involve reviewing example scenarios, and team leads will walk through the actions they would take if the scenario actually takes place. Major incident refresher training will help staff become aware of their responsibilities during these situations so they can confidently act to quickly bring these situations to a successful resolution.

### A More Resilient Health Care System

This viewpoint focuses primarily on the ways ECU Health took targeted actions to mitigate an event that could have been catastrophic for the organization. It is equally important to consider opportunities to make the health care system more resilient as a whole. One such proposal would be to shift the culture toward a position of resilience that ensures clinical workflows are carefully considered and changes are thoroughly tested in simulated environments before being implemented to limit the risk of major interruptions [18]. While such a recommendation may be challenging to implement at scale, if successful, this would go a long way toward shoring up confidence and trust in these vendors and demonstrating their commitment to preventing major disruptions in the future.

Major health IT disruptions also highlight important social and economic inequalities that are pervasive in health care. As compared with smaller institutions, larger organizations often have the capital to invest more heavily in cybersecurity prevention measures and the ability to mobilize resources more quickly when a disruption event occurs [19]. This mismatch can further exacerbate existing disparities in care by causing additional delays in delivery. A truly resilient health care system would ensure that these gaps are closed.

## Conclusions

Major IT disruptions have the potential to adversely impact the delivery of patient care services. In addition, the CrowdStrike outage is estimated to have caused US $1.9 billion in losses to the health care industry [20]. This viewpoint emphasizes the critical role clinical informaticists can play in supporting health care organizations in times of crisis. By having a well-defined incident response plan, organizations can mobilize resources quickly to restore services and ensure clinicians have the tools they need to deliver care and mitigate many of the potential financial harms.

Widespread health care IT disruptions in geographically dispersed rural health care systems pose challenges for timely remediation and continuity of clinical operations while ensuring patient safety. Following the CrowdStrike outage in the summer of 2024, ECU Health relied on coordination and collaboration between multiple teams within IS, including a network of clinical informaticists integrated across practice settings. Lessons learned included the need for continuous commitment and refinement of a systemwide approach to incident response plans, clear communication channels with contingency plans if communications are disrupted, and the value of regular training, such as drills or tabletop exercises, for incident response teams. Through the diligence and collaboration of our multidisciplinary teams, IS teams, and CIs, ECU Health demonstrated resilience and adaptability to ensure we are better prepared for future challenges and remain committed to delivering uninterrupted, high-quality patient care.

## Abbreviations

BSOD: blue screen of death
EHR: electronic health record
EUS: end-user support
HIPAA: Health Insurance Portability and Accountability Act
IS: Information Services
SITREP: situation report
